# Supplementary material for: Are carbon emissions trading and green financial instruments synergistic? -Comprehensive quantitative research based on content analysis
Source: PLoS One. 2024 Mar 7;19(3):e0298601. doi: 10.1371/journal.pone.0298601 (PMC10919601; doi:10.1371/journal.pone.0298601)
Supplement: S1 Data — (ZIP) [file pone.0298601.s001.zip › Data/地级市Z_F工作报告.zip!/╔╜╬≈/╜·│╟╩╨/└┤╫╘╣1⁄2╓┌║┼í╛┬φ┐╦╩2╛▌═°í┐_2014.docx]

各位代表：

现在，我代表市人民政府向大会报告工作，请予审议，并请市政协委员和列席人员提出意见。

一、2013年工作回顾

　　过去的一年，面对经济下行压力，在中共晋城市委的正确领导下，在市人大的有力监督和市政协的大力支持下，市政府认真贯彻落实党的十八大、十八届二中、三中全会和习近平总书记一系列重要讲话精神，坚持“稳中求进”总基调，围绕“一争三快两率先”总战略，以提高发展质量和效益为中心，以转型综改试验区建设为抓手，稳增长、调结构、促改革、惠民生，经济社会发展呈现出稳中有进的良好态势。生产总值完成1031.8亿元，同比增长9.3%；规模以上工业增加值增长12%；固定资产投资完成837.7亿元，增长27.9%；公共财政预算收入完成94.6亿元，增长14.1%；社会消费品零售总额完成297.1亿元，增长14%；外贸出口总额完成2.6亿美元，增长5.5%；城镇居民人均可支配收入完成23250元，增长10%；农民人均纯收入完成9026元，增长12.3%。市六届人大四次会议确定的各项目标任务基本实现。

——政策调控作用明显，产业结构进一步优化。面对错综复杂的经济形势，市政府及时出台保煤保价10条措施、助推实体经济12条意见、加快园区建设8项办法、推动中小微企业发展等一系列政策措施，促进了全市经济平稳运行。煤炭行业增加值增长13.9%，非煤行业增加值增长7.3%。其中，电力行业增长1.7%，煤层气行业增长18.3%，煤化工行业增长3.6%，装备制造业增长13.9%，旅游总收入增长30%。民营经济增加值完成662.5亿元，增长17.2%。银行业贷存比达51.1%，同比提高了3.9个百分点，规模外融投资达366.4亿元，增长124.8%。62个工业转型项目累计完成投资112.3亿元。总投资194亿元的34个重点商贸物流项目正在加快建设。

——项目建设步伐加快，招商引资势头强劲。扎实开展“项目推进年”活动，全市固定资产投资完成837.7亿元，其中，第一产业投资完成59.4亿元，增长1.1倍；第二产业投资完成391.4亿元，增长7.7%；第三产业投资完成386.9亿元，增长47%。全年签约招商引资项目286个，资金到位606.7亿元。当年签约、当年落地项目超过1/3；非资源类占比达85%，10亿元以上项目超过75%。“六位一体”年度目标全部超额完成，固定资产投资增速连续两年居全省第一。继续加强与长三角、环渤海等经济发达地区的联系。与中海油、中国恒天集团、南非卡卡杜市等签订了战略合作协议。组团参加了第八届中博会、农博会等大型展会。举办了第二届中国（晋城）太行山国际文化旅游节。晋城经济开发区升级为国家级开发区，出口监管仓库、保税仓库顺利通过海关验收。

——改革力度不断加大，综改建设初见成效。制定2013-2015年转型综改《实施方案》和2013年《行动计划》。全面展开巴公扩权强镇、西北片区改造等10个综改试点。启动国家低碳城市创建工作，出台“飞地经济”行动计划，制定《气化晋城实施方案》。积极“竞逐中原”，与郑州铁路局、济源市、焦作市签署了合作协议，与中原经济区10市开通了旅游直通车。加大简政放权力度，在全市行政机关推行了服务承诺、首问责任、限时办结三项制度和建设项目联合办理、重点项目审批“绿色通道”、行政审批首席代表制三个办法。对巴公综改试点、西北片区改造下放了涉及9个部门的57项审批权限。组建煤炭和煤层气工业管理局，下发《深化户籍管理制度改革实施意见》，成功申报全国循环经济示范创建市、智慧城市试点市和新能源汽车示范城市。

——城乡统筹扎实推进，生态环境治理成效明显。中心城市投资近百亿元，启动了包括17条城市道路的41项城建重点工程。新增集中供热面积140万平方米，新增市区煤层气用户1.17万户。西北片区实施整体改造，兰花片区、金村片区、北石店片区和金匠工业园区等加快建设。大县城、中心镇和美丽乡村统筹推进。河西镇、町店镇被命名为省级园林城镇。周村、天井关村等17个村列入中国传统村落名录，11个镇村新入选中国历史文化名镇名村。农村新增清洁能源用户3万户。节水型社会试点市建设通过国家水利部验收。新增造林绿化面积15.13万亩，在全省率先实现了国家森林城市创建目标。实施污染减排项目58个，对六大重点耗能行业进行能效对标，6项主要污染物排放量大幅削减。丹河人工湿地建成投运。

——“三农”工作全面加强，农民收入稳定提高。落实强农惠农资金32.4亿元。全力开展“一抗两保”，实现了粮食生产受灾之年仍获丰收，全年粮食总产达18.1亿斤。生猪出栏达165.4万头，位居全省第一。新建现代农业示范园区30家，200亩以上的设施农业园区达到97家。新发展农民专业合作社1108个。加快“一村一品”、“一县一业”和新农村连片示范区建设，实施了街道亮化、移民搬迁、危房改造和农村清洁工程，启动“百企千村”产业扶贫工作。扎实开展干部下乡住村帮扶活动，解决实际问题3930项，村容村貌和农村生产生活条件进一步改善。

——民生事业不断发展，人民生活持续改善。民生支出增长25%，占到全市公共财政支出的83%。太原科技大学晋城校区正式招生，圆了全市人民多年的大学梦。新改扩建幼儿园53所,免除城市义务教育教科书费惠及学生6.71万人。表彰“晋城专家”和“晋城名师”，提高乡村医生工作待遇。连续七届荣获“全国科技进步先进市”称号。城镇新增就业岗位4.23 万个，城镇登记失业率控制在1.53 %以内。城乡居民基础养老金、企业离退休人员基本养老金实现“九连增”。城乡居民殡葬惠民政策全面实施，市社会福利院新楼投入使用。新开工各类保障性住房17908套。继续开展公益演出、周末大剧场、特色文化村镇、农村电视数字化升级改造等文化低保工程。省定五件实事和市定10件实事全部完成。统计、地震、气象、人防、治超、民族宗教、妇女儿童、人口计生、档案史志、外事侨务、老龄、残疾人、红十字、慈善救助等社会事业，都取得了新的成绩。

——社会治理不断加强，发展大局和谐稳定。深入推进精神文明和民主法治建设。大力加强群众性精神文明建设，努力培育和践行社会主义核心价值观。自觉接受人大及其常委会的决议、决定，支持人民政协履行职能，办理人大代表建议和政协提案各361件。加强法治政府建设，扎实开展“六五”普法和政风行风建设。严格执行中央“八项规定”，市级一般性支出压缩23%，“三公”经费下降14.2%。深入严打整治，强化信访和社会矛盾排查调处工作，群众安全感和满意度民意测评中连续两年名列全省第一，连续五届二十年荣获全国综治工作优秀市，三次捧得“长安杯”。全民国防教育更加深入，国防后备力量建设质量不断提升。安全生产总体平稳，全年各类事故死亡人数下降3.4%，工矿商贸企业首次实现3人以上较大事故零发生。

各位代表，在主导产业煤炭形势发生巨大变化、我市结构性矛盾更加凸显的情况下，取得这些成绩来之不易，超过了我们的预期。这是我们认真贯彻执行党中央、国务院和省委、省政府一系列方针政策的结果，是中共晋城市委统揽全局、正确领导的结果，是市人大、市政协有效监督、全力支持的结果，是全市人民辛勤工作、共同努力的结果。在此，我代表市人民政府，向全市人民，向驻市解放军指战员、武警官兵和公安干警，向各民主党派、各人民团体和各界人士，向所有关心、支持晋城发展的同志们、朋友们，表示衷心的感谢和崇高的敬意！

同时，我们清醒地看到：产业结构单一，新兴产业成长不快，经济发展质量效益不高，转型升级任重道远；市场需求不旺，煤炭量价双跌，经济运行下行压力增大;体制性、机制性改革创新不够，经济增长的内生动力和活力不足；科技驱动、资本市场、生态环境、社会治理、民生事业、财政增收和安全生产等方面仍然存在很多问题和不足。

我们还清醒地看到：一些政府机关和工作部门形式主义、官僚主义等还不同程度地存在。有的以会议落实会议，以文件落实文件，工作不细不实不到位；有的遇事推诿扯皮，缺乏担当意识，有利的事情争着干，没利的事情“踢皮球”；有的对上级交办的任务找客观原因顶着不办，对群众要办的事情找各种理由拖着不办；有的求稳怕乱，回避矛盾，不敢担当，不愿负责，甘当“太平官”等等。对此，我们必须高度重视，认真加以整改和解决。

二、2014年工作安排

2014年是全面贯彻落实党的十八届三中全会精神的开局之年，也是实现“十二五”规划目标的关键之年。今年政府工作的指导思想是：全面贯彻落实党的十八大和十八届三中全会精神，坚持“稳中求进、改革创新”的总基调、“一争三快两率先”的总战略、“只争朝夕、一抓到底”的总要求，把改革创新贯穿于经济社会发展全过程各方面，统筹推进工业新型化、农业现代化、新型城镇化、城乡生态化，更加突出改革红利和市场活力，更加突出产业转型和创新驱动，更加突出民生改善和环境优化，更加突出政府职能和干部作风转变，促进全市经济持续健康发展、社会和谐稳定。

今年我市经济社会发展的主要目标是：地区生产总值增长10%左右，固定资产投资增长25%，社会消费品零售总额增长15%左右，公共财政预算收入增长10%，城镇居民人均可支配收入增长11%左右，农民人均纯收入增长11%以上。城镇新增就业岗位2.5万个，城镇登记失业率控制在4.2%以内。居民消费价格涨幅控制在3.5%左右。万元地区生产总值综合能耗下降3.5%，万元地区生产总值二氧化碳排放量下降3.7%。市区 PM2.5下降4%。

实现上述目标，要重点做好八方面工作：

（一）全面深化改革开放

改革开放是推动转型跨越的强大动力。要在中央、省顶层设计的大框架下，聚焦改革重点，推动改革实践，以改革的领先实现发展的率先。

开展“转型综改攻坚年”活动。认真落实好省定各项综改任务，解放思想、先行先试、立说立行，全力推进我市三年综改实施方案和2014年行动计划。以巴公扩权强镇、西北片区改造、金村低碳示范区为重点的“1+9”综改试点要先行一步，扎实推进，力求取得突破性成果。低碳城市、飞地经济、气化晋城、城乡统筹、竞逐中原等综改的重大事项要细化目标，强化措施，落实责任，确保取得实质性进展。

深化重点领域改革。深化财税体制改革，改革预算管理制度，整合规范各种专项转移支付，盘活存量资金，提高财政资金使用效益。深化国有资产经营管理体制改革，健全公司法人治理结构，优化国有资产经营能力。加快金融改革，鼓励各类金融创新，做实做强融资平台，城区、泽州、阳城农村信用社完成改制。优化政府组织结构，完成市县政府机构改革。按照责权利相统一、建设管理相分离的原则，推进城市建设和管理体制改革。按照全省“一核一圈三群”的总布局和综改要求，积极稳妥科学地启动区域规划调整。

努力提高对外开放的水平和质量。坚持招商引资与招才引智并举，对外开放与对内搞活并重，市场、企业、政府互动，大中小微企业同行的思路，深化同重点地区、重点企业全方位合作对接。围绕全市产业优势和定位，充分发挥骨干企业和商会的作用，加大招商引资力度，以更加开放的理念，在更大空间、更高层面上优化配置资源，提高招商引资的质量和效率。加快高平和阳城“飞地经济”建设。特别要加快与中原经济区的深度融合，在产业转移、交通体系、商贸旅游等方面全方位开放、多层次协作，力争把我市建设成为中原地区人流、物流、信息流的聚集区。积极推进晋城海关、商检和综合保税区建设，为扩大开放提供保障。

各位代表，晋城是在改革开放大潮中诞生和成长起来的新兴城市，当前正处于转型升级的重要历史节点。不深化改革，不扩大开放，不转型升级，就没有出路，就没有前途。我们只有凝聚正能量，搞好大合唱，坚持全面改革不动摇，坚持先行先试不懈怠，坚持转型跨越不松劲，才能继续谱写出晋城转型跨越的新篇章！

（二）加快产业转型升级

晋城是以煤炭为主导产业的资源型城市。打造晋城经济的升级版，必须紧紧扭住产业转型的牛鼻子。产业转型升级的方向，就是以煤为基、多元发展，加快产业结构的调整和优化；转型升级的重要途径，就是通过创新驱动，激活生产要素，提高质量效益；转型升级的中心任务,就是改造提升传统产业，培育壮大新兴产业，大力发展第三产业，通过产业集聚集群，提升晋城发展的核心竞争力。

继续做好煤炭这篇大文章。晋城是典型的煤炭经济。今年的煤炭形势要比去年更加严峻。要密切关注市场变化，底线思维，趋利避害，加强监测和调控，着力在盘活存量、提升质量、防范风险上下功夫，着力在安全、生产、建设、营销等各个环节上下功夫，努力保障煤炭经济平稳健康运行，巩固好经济的基本面。重点抓好里必、沟底、玉溪、东大、龙湾等现代化骨干煤矿建设，加快整合矿井建设进度，形成有效产能1575万吨/年。全年煤炭产量同比增加500万吨以上。大力发展煤商贸、煤物流、煤机装备、煤金融、煤环保等特色关联产业，构建我市煤炭产业新体系。

加快国家综合能源基地建设。充分发挥我市煤炭、煤层气比较优势，着力构建“煤、电、气、化”综合能源基地，创造晋城经济新的竞争优势。抓住低热值煤发电项目省核准机遇，力争胡底低热值煤发电项目通过核准，争取高平煤电气化一体化项目早日开工，启动国投热电厂二期项目。鼓励煤电、煤化联盟，推进煤电一体化、煤气一体化产业链，提高低热值高硫煤就地转化率，实现从煤炭一个优势向煤电气化综合优势转变。围绕沁水煤层气总部基地建设目标，加快晋城煤层气集输总站、国家煤层气重点实验室、煤层气交易中心、煤层气装备制造和煤层气综合利用等重点项目建设。

打造晋城工业经济升级版。依托传统产业基础，做好转型升级规划，突出重点，分业施策，明确各类产业发展的方向和路径。下决心重组整合铸造、化工、丝麻产业，通过兼并重组、技术创新、全产业链条模式和循环经济等措施，做强龙头企业，做大特色产业，形成产业集群。化工产业，加快新型气化技术攻关和示范项目建设，以煤基新材料、煤基新能源为方向，开发精细化、差异化的高端化工产品，实现由化肥单打到肥化并举,再到现代精细化工产业链条和循环经济迈进。重点抓好晋煤华昱高硫煤洁净利用化电热一体化项目、天泽乙二醇和兰花钠米碳酸钙、己内酰胺等重点项目。装备制造业，加快泽州铸造园区建设，重组泽州、高平铸造企业，形成铸造—机加工—装备制造产业链条。重点抓好福川精密铸件、清慧新型材料高端结构件铸造、金鼎煤机、富士康、力宇燃气动力装备、中道能源新能源动力汽车等先进装备制造项目，推动晋城铸造向晋城制造、晋城创造飞跃。围绕支柱产业和新兴产业，加强与高等院校、科研院所合作，建立形成紧密的产业技术创新联盟，力争无烟煤、高硫煤深加工转化项目进入国家级或省级重大科技攻关行列，争抢晋城煤化产业新的制高点。大力引进各类领军人才特别是创新团队，用最大的决心、最优的服务、最好的环境吸引支持高层次人才来晋城投资创业。坚决破除观念性、体制性和环境性障碍，认真落实促进民营经济发展的各项政策和措施，打破“玻璃门”、“弹簧门”，全力推动民营经济、混合所有制经济和中小微企业快速发展。

大力发展现代服务业。积极推进联通云计算、微软外包基地项目落地，以龙头项目带动发展新的产业形态。加快会展中心、上海国际汽车城、兰花国际物流园等项目建设。以陵川县为重点，整合做强南太行旅游资源，叫响“太行山”旅游品牌。保护性修缮程颢书院，启动炎帝陵和沁河古堡群修复开发，彰显晋城历史记忆和文化内涵。积极发展电子商务、研发设计、家政服务、育幼养老、医疗保健等新兴服务业。

切实加大投资力度。按照全省“项目见效年”要求，今年要确保固定资产投资1047亿元和重点工程投资872亿元的年度目标，确保各县（市、区）、开发区都要有50亿元至100亿元的招商引资大项目、好项目。实现两个“确保”，一要抓好园区“五规合一”。加快北留周村煤电化工业园、金匠先进装备制造工业园、高平新能源科技创新园、泽州铸造产业工业园、阳城陶瓷产业工业园等的基础设施建设，真正把园区建成产业集聚、企业集群的主要载体。市里建立专项资金，以奖代补，竞争性支持各县（市、区）园区建设。二要创新金融模式，激发资本市场，学会用别人的钱办自己的事。整合煤炭等各类优质资源，做大做强市县两级融资平台，做优做活各类工程投融资机制，真正做到资源资产化、资产资本化、资本市场化，为招商引资、基础建设、产业发展、公共服务提供支持。加快投资增速，要发挥好高平、泽州、沁水、阳城、开发区等重点县域和晋煤、兰花、晋能、天泽、富士康等骨干企业的带动作用，发挥好高沁、阳济高速公路和晋阳一级路等重大基础设施建设的拉动作用。要成立专门机构，切实抓紧推进铁路、机场建设的前期工作。

（三）切实改善生态环境

生态环境是晋城的优势和品牌，必须全力护好这张牌，打好这张牌。要以建立生态文明制度体系为目标，以建设“气化晋城”、“绿化晋城”、“净化晋城”为重点，加快美丽晋城建设。

“气化晋城”。落实《气化晋城实施方案》，加快推进煤层气高压集输系统建设，争取用两年时间实现6个县（市、区）、重点工业用户、重点旅游区、重点乡镇等四个“全覆盖”，实现主城区100%、工业园区100%、县城90%、乡镇和主要村庄80%的气化率目标。

“绿化晋城”。坚持生态产业衔接，城乡一体统筹，一、三产业互动的原则，按照整体规划、多方投入、生态优先、绿道串行的思路，力争用五年的时间，建设集产业、生态、交通、旅游观光、休闲娱乐、美丽乡村、文化传承等为一体的396平方公里的环城森林公园。主要包括白马寺山至丹河、龙王山至丹河两条生态廊道和城东丹河东岸、城西玉屏山、城北巴公莒山、城西南晋普山、城东南桑坪等环城森林公园。改造提升赵树理公园、玉龙潭公园。新建白水河公园、龙湾公园、北石店公园、城市西入口绿化、水陆院庙会文化广场和五处街头绿地。完成国省营造林8万亩，植树600万株，中幼林抚育3万亩。

“净化晋城”。打好“十组拳”，开展改善环境质量攻坚行动。两年内关停城市规划区域内的古书院、王台铺、天安广利、北岩等煤矿。年内完成中石化油库、一化搬迁。实施煤改气、油改气等综合治理，逐步取缔城市燃煤锅炉，淘汰黄标车。启动沁河、丹河生态修复工程，开工建设沁河端氏—润城段、丹河高平城区段、泽州金村段生态治理工程。加快金匠、北石店、重点乡镇污水处理厂建设和阳城、沁水污水处理厂提标步伐。加快市区西河上游、花园头河、白水河治理改造步伐，逐步打造城市生态景观。

狠抓电力、钢铁、化工、水泥等重点行业节能减排和化解产能过剩。严格执行环评、能评制度，加大对高耗能、高污染企业和项目的监管力度。抓住我市国家循环经济示范创建市、国家低碳城市试点市和新能源汽车示范城市的机遇，完善循环经济发展机制，推行“碳评价”制度，积极开发和使用绿色清洁能源，全面推进经济社会的绿色发展、循环发展、低碳发展。

各位代表，“有了温饱更要环保”，“有了小康更要健康”。我们要以对人民负责、对未来负责、对子孙后代负责的态度，用壮士断腕的决心和勇气来改善环境质量，努力使人民群众呼吸上清新的空气、喝上清洁的水、吃上安全的食品、享受上舒适的环境！

（四）大力推进新型城镇化

认真贯彻中央城镇化工作会议精神，坚持“以人为本、优化布局、生态文明、传承文化”的基本原则，切实提高新型城镇化质量和水平。

加大中心城市建设力度。以花园城市、智慧城市和全国文明城市为目标，三年投入200亿元，山水园林路统筹推进，迎接建市30周年。按照“拓展、改造、提质”的总体思路，加快西北片区和兰花片区改造速度，三年见成效，五年大变样，实现城市品质总体提升。以“一园两环、三纵三横”为重点，建设城市主框架。“一园”就是396平方公里的环城森林公园；“两环”就是207国道和陵沁线连接构成的内环和两路改线外移后连接形成的外环两条快速通道；“三纵三横”就是市区的纵道泽州路、文博路、景西路，横道书院街(太岳街)、凤台街、白水街。开工建设书院街、景西南路、白水街西段和207国道改线、陵沁线改线等主城区道路重点工程，逐步打通市区“断头路”，实现“大循环”。开工建设金凤路、金石路、畅安路南段、太岳街东段等道路，打通主城区和片区连接线，实现六区联动。出台相关政策，加快城中村改造，全力打造花园头河、丹河公园等城市新地标。

抓好大县城和产业片区建设。在加快城镇化建设中，要突出大县城主阵地和产业片区的主战场作用。大县城建设要以扩大规模、增强实力、完善功能、体现特色为基本路径，积极引导生产要素、优势资源向大县城集中，力求在基础设施、公共服务、土地利用、产业发展、融资渠道上先行先试、破解难题，增强县城对县域经济的带动作用和吸纳集聚人口的能力。以产业为支撑，以园区为载体，修路治水栽树，兴业聚人扩镇，集中打造巴公、马村、嘉峰、北留-周村、南村、米山等各具特点的产业片区，推动农民就地城镇化。泽州县要抓住机遇，集中全力，扎实推进城乡统筹金村低碳示范区建设。

提升城市管理水平。坚持建管并重，标本兼治，提高城市管理能力和执法水平。加强城市规划的实施管理，维护城市规划的权威性和严肃性，严格查处违法建设。加大市容环境整治力度，重点整治市区乱停乱放、乱搭乱建、乱摆摊点、占道经营等群众反映强烈的问题。要认真落实缓解城市交通拥堵的实施意见，改善城市交通环境。引导和培养广大市民爱我晋城、热爱家园的城市意识。积极利用现代信息技术，推进“智慧城市”建设。

（五）进一步做好“三农”工作

认真落实中央一号文件精神，坚持把“三农”问题作为重中之重，全面深化农村改革，强化农业、惠及农村、富裕农民。

扎实推进农村人居环境改善。继续实施以农村气化、环境整治、生态绿化、污水处理为主要内容的农村清洁工程，以中心镇为重点，提升农村公共服务水平，推动农民生产生活方式转变。统筹农村危房改造、采煤沉陷区治理、移民搬迁、扶贫开发、新农村建设和城镇化建设，集中力量推进农村环境综合整治，增加山水底色、人文气息、现代色彩，加快美丽乡村建设。

大力发展现代特色农业。毫不放松抓好粮食生产，保护和调动农民种粮积极性。以“一村一品、一县一业”为切入点，打造畜牧、蚕桑、蔬菜、干鲜果、小杂粮、中药材、食用菌、苗木花卉等特色农业优势产业，努力形成晋城工厂化食用菌，高平、泽州生猪，阳城、沁水蚕桑，陵川中药材，沁水苗木花卉，丹河、沁河流域设施蔬菜的具有晋城特色的现代农业产业集聚基地。

努力增加农民收入。认真落实粮食补贴、良种补贴、农资价格综合补贴、农机具购置补贴等强农惠农政策。鼓励发展专业大户、家庭农场、农民合作社、龙头企业等新型农业经营主体。坚持稳定土地承包关系，依法保障农民对承包地占有、使用、收益、流转及承包经营权抵押、担保权能。围绕农民增收加快农村土地流转，今年要出台具体实施办法，做活土地流转，确保转得出、用得好、补偿合理。

抓好农村基础设施建设。加快“井”字型大水网建设，重点抓好郭壁供水改扩建、下河泉北留供水续建、杜河提水、任庄水库、磨河水库、张峰水库阳城供水、张峰水库泽州供水、张峰水库高平供水东延、市区河道清淤等10项水利工程，提高水资源高效配置和安全保障。加大土地开发，加强耕地保护。

加大扶贫力度。创新扶贫开发模式，提高扶贫开发效果。加强新型职业农民培训，增强贫困人口自我发展能力。依托产业确保贫困户脱贫致富，集中晋煤、兰花、晋能、天泽等骨干企业重点支持陵川县产业扶贫。

各位代表，小康不小康，关键看老乡。我们要决心不变，力度不减，持之以恒，一定让农业强起来、农村美起来、农民富起来！

（六）着力保障和改善民生

优先发展教育。加快推进义务教育优质均衡发展，强化公办学校尤其是农村义务教育薄弱学校标准化建设的投入机制和长效机制，逐步缩小区域、城乡、校际差距，让我们的每一个孩子不要输在人生的起跑线上。加快发展现代职业教育，续建太原科大晋城校区，规划建设金村教育园区。加强教师队伍建设，研究提高教师工资待遇。提升特殊教育，抓好学前教育、民办教育和继续教育，促进各级各类教育全面协调发展。今年开工建设颐翠中学、郝匠小学、书院小学等8所市区中小学校。

兜住底线民生。认真落实中央、省加强和改进最低生活保障工作的意见，制定出台实施细则。提高城镇居民、新农合财政补助标准和实际报销水平，提高农村五保户集中供养、分散供养补助标准，建立重度残疾人护理补贴和贫困以及重度残疾人生活补贴制度，对贫困残疾人进行康复救助，继续加强养老服务机构建设，确保我市社会福利水平位居全省前列。

做好热点民生。大力开展职业技能培训，实施更加积极有效的就业政策。搞好粮、油、肉、菜的生产和储备，降低流通环节费用，发挥价格调节基金作用，完善社会救助和保障标准与物价上涨挂钩的联动机制。加大商品房土地供给，扩大住房公积金覆盖范围，新开工建设各类保障性住房15000套，让更多的群众住上价廉舒适的新房。继续深化医药卫生体制改革，积极预防重大传染病发生，提高重大突发公共卫生事件处置能力，健全重特大疾病医疗保险和救助制度，鼓励和引导社会资本开办医疗机构, 建设健康晋城。

加快发展科技、文化、体育等社会事业。继续把推进科技创新放在突出位置，加强科技队伍建设，建立以企业为主体的技术创新体系。有序落实“单独两孩”政策，促进人口长期均衡发展。深化文化体制改革，继续实施文化建设“六大工程”，加强基层文化基础设施和人才队伍建设，推进上党（晋城）省级文化生态保护试验区建设，开工建设公共文化活动中心，推进文化强市战略。广泛开展全民健身运动，促进群众体育和竞技体育全面发展。大力发展老龄、残疾人、慈善、红十字事业，继续做好地震、气象、人防、对台、档案史志、民族宗教、外事侨务、文物保护、广播电视、妇女儿童等各项工作。

各位代表，不断提高人民群众的物质文化生活水平是我们一切工作的出发点和落脚点，为人民群众谋福祉是我们的不懈追求。新的一年，我们要投入更多的人力物力财力，把各项社会事业发展好，把事关群众切身利益的事情办好，让人民群众生活得更加富裕、更加幸福、更有尊严！

（七）抓好社会治理创新

加强民主法治建设。做好第十届村委会换届选举工作，提升基层自治组织的管理能力。深入推进政务公开、村务公开、厂务公开，保障人民群众的知情权、参与权、表达权、监督权。围绕法治晋城目标，大力推进严格执法、公正司法、全民守法，全面提高社会治理水平，着力营造规范有序、公平公正的法治环境。

加强精神文明建设。弘扬晋城精神，提高文明素养，争创全国文明城市。大力培育和践行社会主义核心价值观，加强社会公德、职业道德、家庭美德、个人品德的宣传教育，倡导勤劳节俭、遵德守礼、孝老敬老风尚，推进诚信建设制度化、志愿服务制度化。进一步加强和改进未成年人思想道德建设工作。

加强和创新社会治理。完善信访工作和矛盾调处机制，健全基层社会服务网格化管理体系，有效预防和化解社会矛盾。加强食品药品安全监管，保障人民群众饮食用药安全。加强应急管理和防灾减灾能力建设，妥善应对各类突发事件。依法加强互联网管理工作，确保网络和信息安全。推进社会治安综合治理，不断引深平安创建活动。继续加强国防动员、国防教育和国防后备力量建设，积极开展双拥工作，促进军民融合发展。

切实抓好安全生产。安全生产是民生，是最大的民生；是责任，是比泰山还重的责任；是红线，是任何时候任何地方都不可触碰的高压红线。要全面贯彻落实党中央、国务院和省委、省政府关于安全生产的一系列指示精神，严格落实安全生产政府和企业两个主体责任，建立健全隐患排查治理体系，全面深化安全专项整治，严厉打击各种违法违规行为，减少一般性事故，坚决防止重大事故发生，保持安全生产形势持续平稳。

（八）努力打造法治政府和服务型政府

做好今年各项工作，必须以扎实开展好第二批党的群众路线教育实践活动为动力，以改革创新精神全面加强政府自身建设，时刻牢记最好的工作是只争朝夕，最好的落实是一抓到底，最好的公信是说到做到，不断增强政府的公信力和执行力。

加快转变政府职能。强化政府公共服务、市场监管、社会管理、环境保护等职责。以职能转变为核心推进“流程再造”，深化行政审批制度改革，按照“集中、便民、提效”的要求，落实“两集中、两到位”，最大限度地简政放权。健全依法科学民主决策制度，提高政府决策水平，规范政府行政行为。推进机构编制管理科学化、规范化和法制化。

提升行政效能。强化行政监察，严格行政问责。合理调整考核内容和权重，完善综合绩效管理与考核评价机制，奖优、治庸、罚劣。着力解决群众反映强烈的突出问题，强化干部问责、问绩、问效措施，使想干事的有机会，能干事的有舞台，干成事的有位置。加强公务员队伍建设，提升管理能力、服务能力和执行能力，确保政令畅通、令行禁止。

全面推进依法行政。强化法治思维，践行法治理念。建立政府法律顾问制度，完善规范性文件、重大决策合法性审查机制，保障法律的正确实施。严格执行人大及其常委会的决议决定，主动接受政协民主监督，认真办理人大代表议案、建议和政协提案。广泛听取民主党派、工商联、无党派人士意见，充分发挥工会、共青团、妇联等人民团体作用。支持法院、检察院依法独立公正行使职权。加强行政监察和审计监督，虚心接受社会监督和舆论监督。深化政务公开，完善新闻发布制度，让权力在阳光下运行，让人民监督权力！

努力建设清廉政府。健全惩治和预防腐败体系，坚持用制度管权管事管人。严格执行党风廉政建设责任制和廉洁从政各项规定，认真执行“八项规定”和《党政机关厉行节约反对浪费条例》，进一步压缩“三公”经费，规范公务接待，禁止新建楼堂馆所，财政供养人员只减不增。加强预算编制和政府债务管理，强化财政资金使用、重大项目投资、工程招投标、政府采购等重点领域监管，严肃查处各类违法违纪案件，做到干部清正、政府清廉。

在做好以上各项工作的同时，今年还要办好十件实事：一是改造农村困难家庭危房1393户；二是深入推进乡村清洁工程；三是移民搬迁4200人；四是改造农村幼儿园15所；五是培训新型职业农民1万名；六是新增集中供热面积160万平方米；七是完成农网改造升级工程；八是完成3000名残疾人康复救助工程；九是提高城乡居民最低生活保障标准；十是调整城镇职工居民基本医疗保险报销比例和增加医疗保险门诊慢性病病种。

各位代表，人民创造历史，发展全凭实干。让我们紧密团结在以习近平同志为总书记的党中央周围，在中共晋城市委的坚强领导下，紧紧依靠全市人民，齐心协力，埋头苦干，奋力开创转型跨越新局面，为实现“一争三快两率先”战略目标而努力奋斗！
